# A Case of Sublingual Dermoid Cyst: Extending the Limits of the Oral Approach

**DOI:** 10.1155/2012/634949

**Published:** 2012-09-29

**Authors:** Nobuo Ohta, Tomoo Watanabe, Tsukasa Ito, Toshinori Kubota, Yusuke Suzuki, Akihiro Ishida, Seiji Kakehata, Masaru Aoyagi

**Affiliations:** Department of Otolaryngology, Head and Neck Surgery, Yamagata University Faculty of Medicine, 2-2-2 Iida-nishi, Yamagata 990-9585, Japan

## Abstract

We present the case of a dermoid cyst with an oral and a submental component in a 21-year-old Japanese woman who presented with complaints of a mass in the oral cavity and difficulty in chewing and swallowing solid foods for about 2 years. MRI shows a 55 × 65 mm well-circumscribed cystic mass extending from the sublingual area to the mylohyoid muscle. Under general anesthesia and with nasotracheal intubation, the patient underwent surgical removal of the mass. Although the cyst was large and extending mylohyoid muscle, intraoral midline incision was performed through the mucosa overlying the swelling and the cyst was separated from the surrounding tissues with appropriate traction and countertraction and successfully removed without extraoral incision. Oral approach in surgical enucleation is useful procedure to avoid cosmetic problems in large and extending mylohyoid muscle cyst.

## 1. Introduction

Sublingual epidermoid and dermoid cysts are benign lesions encountered throughout the body, with 7% occurring in the head and neck area and 1.6% within the oral cavity [1–5]. They represent less than 0.01% of all oral cavity cysts [6–9]. The pathogenesis of midline cysts of the floor of the mouth is not well established, and dysontogenetic and thyroglossal anomaly theories have been suggested [1–9]. In fact, dermoid cysts occur primarily in the oral cavity, and the most common location in the head and neck is the external third of the eyebrow [1–9]. Dermoid cysts generally present with slow and progressive growth, and even if they are congenital, they are possible in the second or third decade of life [7–9]. The treatment of dermoid cysts of the floor of the mouth is surgical; the approach can be either intraoral or extraoral, depending on the localization and size of the mass. Cysts are classified into three types by localization: (1) sublingual, (2) submental, and (3) submandibular cysts. Oral approach is usually applied for small sublingual cyst. The extraoral incision is preferred in submental and large sublingual cysts. Dermoid cysts usually present early in life as asymptomatic masses; they may reach a large size and involve more than one anatomical area, including that near the hyoid bone [1–9]. Such a swelling on the floor of the mouth can occasionally cause serious problems with swallowing and speaking [1–9]. Here, we outline a case of giant sublingual dermoid cyst in a 21-year-old woman that was successfully removed by oral approach without extraoral incision. 

## 2. Case Report

**Figure 1:**
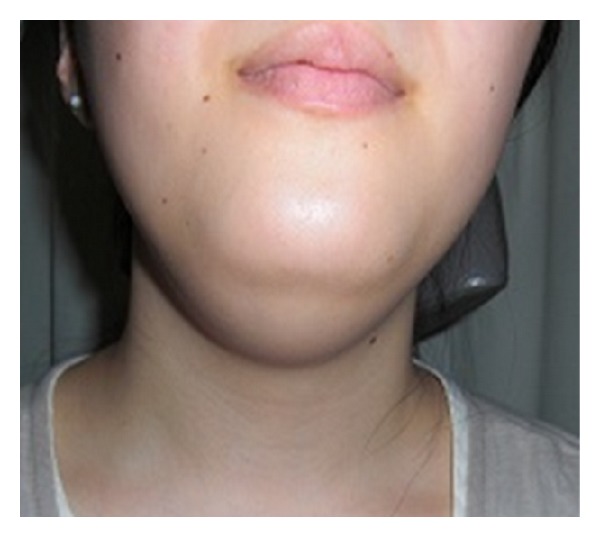
Clinical pre-operative presentation of a submental swelling.

**Figure 2:**
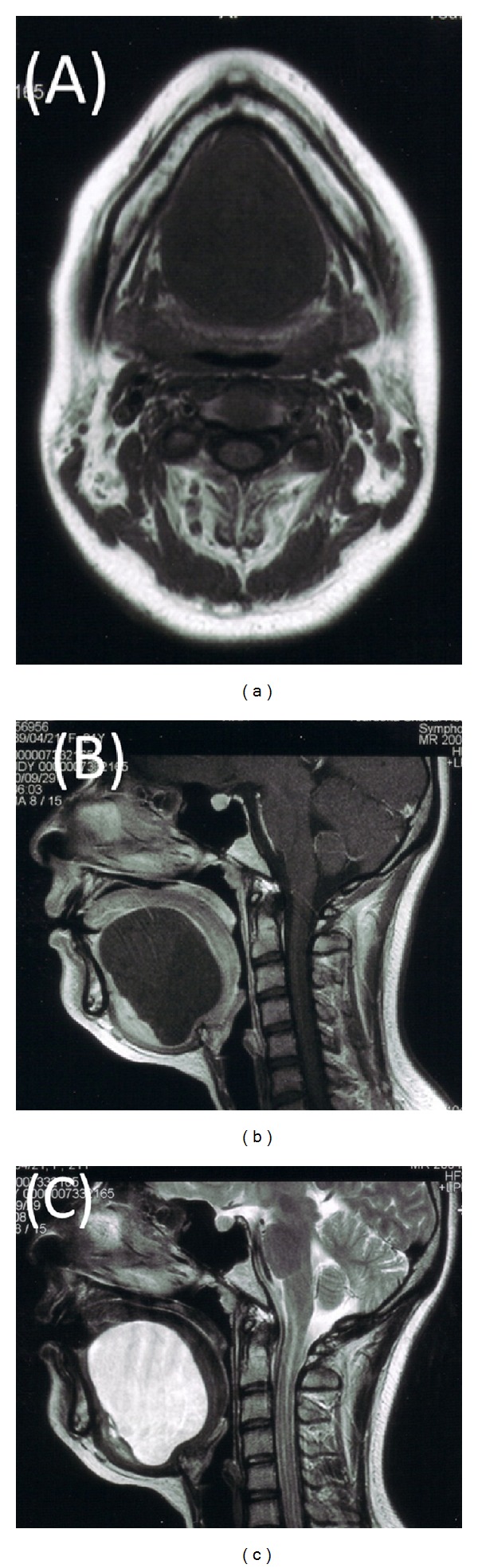
(a) Axial T1-weighted magnetic resonance imaging (MRI) shows a sharply circumscribed cystic mass. (b) Sagittal-T1 weighted MRI shows an 55 × 65-mm well-circumscribed cystic mass extending from the sublingual area to the mylohyoid muscle. (c) Sagittal T2-weighted MRI shows an high-intensity mass extending inferiomedially to the genioglossus muscle and inferiorly to the mylohyoid muscle.

**Figure 3:**
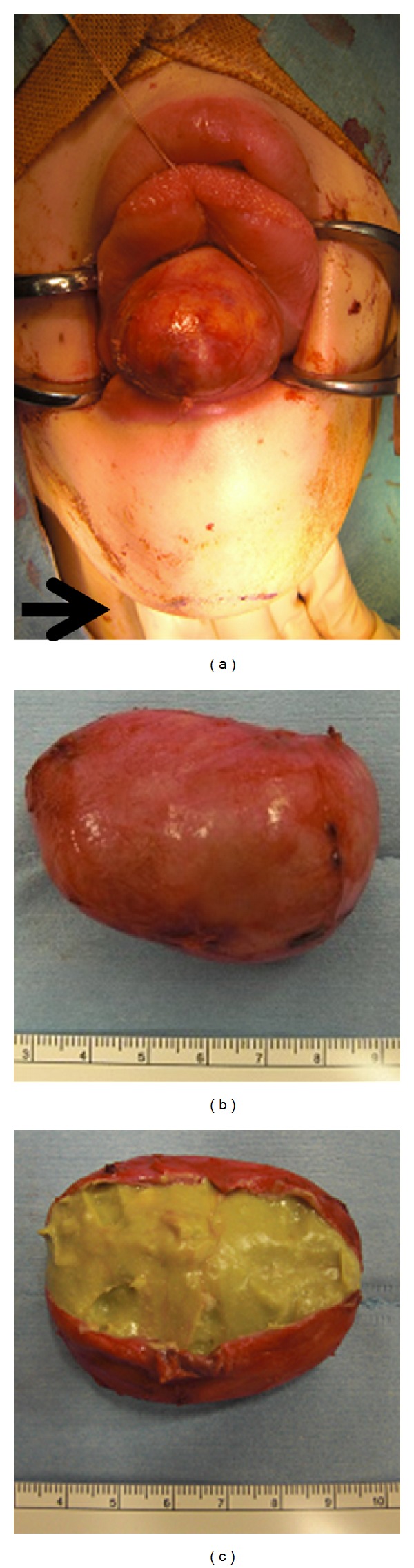
(a) Intra-oral midline incision from the base of the tongue to the floor of the mouth, providing exposure of the lesion. (b) Following removal, the specimen measured approximately 5.5 × 5.6 × 4.5 cm. (c) Photograph of the dissected cyst shows cheesy, solid material.

**Figure 4:**
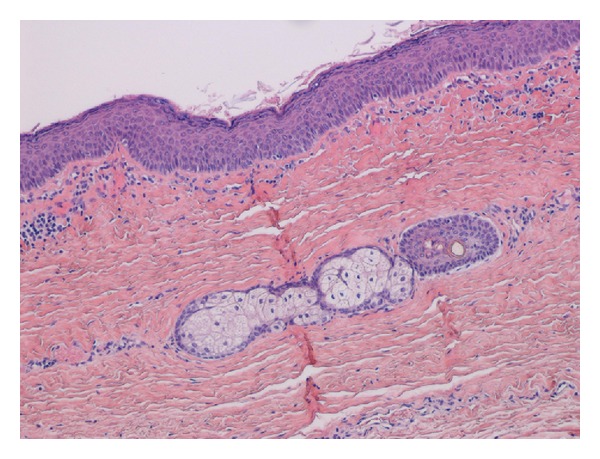
Pathological examination showed orthokeratinized stratified squamous epithelium with a flat epithelial-connective tissue interface lining the cystic cavity, sebaceous glands, and hair, along with copious sebaceous material. (Hematoxylin and eosin staining, original magnification ×200.)

A 21-year-old Japanese woman was referred to our otolaryngology department with the chief complaint of a swelling below the tongue producing difficulty in chewing and swallowing of solid foods for about 2 years. Examination revealed the presence of a solitary midline swelling in the sublingual region measuring 6 × 5 cm. It was nontender, fluctuant, soft, and nonmobile, and the overlying mucosa showed no secondary changes. There were no inflammatory signs or lymphadenopathy associated with the swelling (Figure 1). Axial and sagittal magnetic resonance imaging showed that tumor had low signal intensity on the T1-weighted image and high signal intensity on the T2-weighted image (Figure 2). Aspiration cytology was performed and revealed a cheesy material containing numerous non-nucleated epithelial cells. The aspiration cytology was very helpful for making a diagnosis of epidermoid or dermoid cyst. The patient underwent surgery under general anesthesia with nasotracheal intubation. An intraoral midline incision from the base of the tongue to the floor of the mouth was used to access the lesion (Figure 3). Special attention was paid to the Wharton's ducts to prevent injury bilaterally. The cyst was completely exposed, and on evaluation partial caudal herniation through the mylohyoid muscle was seen. A combination of sharp and blunt dissection was used to free the cyst with traction and couter-traction, and it was delivered intact per os (Figure 3). The wound was closed in layers and a non-vacuum drain was kept in situ for 24 h. Examination with hematoxylin-eosin staining revealed a cystic lesion with a stratified squamous epithelium lining and a fibrovascular connective tissue capsule covering the cystic lumen (Figure 4). These findings are consistent with a dermoid cyst.

## 3. Discussion

Epidermoid and dermoid cysts of the oral cavity represent less than 0.01% of all oral cavity cysts [1–9]. Histologically, this distinction of the cysts in the floor of the mouth was presented by Meyer in 1955 [10]. The cyst is described as epidermoid when the lining presents only epithelium, dermoid when skin adnexa are found, and teratoid when other tissues such as muscle, cartilage, or bone are present within the cyst [10]. Dermoid cysts of the floor of the mouth are disembryogenetic lesions derived from the entrapment and subsequent growth of epithelial cells during midline fusion between the first and second branchial arches in the third and fourth embryonic weeks [10]. Acquired forms are derived from either iatrogenic or traumatic inclusion of epithelium and skin appendages [8–10]. Dermoid cysts are generally diagnosed in the second and third decades of life but can present at any age. Congenital cysts of ectodermal origin are uncommon in the oral cavity (1.6%), and epidermoid cysts rarely occur there. Midline cysts of the floor of the mouth are painless lesions that swell from the anterior portion of this region. Because these cysts can displace the tongue, patients usually present with dysphagia, dysphonia, and dyspnea; in the case of lower localization they present with a characteristic double chin [1–9]. Anatomically, three different types of dermoid cysts can be distinguished: median genio-glossal (sublingual), median geniohyoid (submental), and lateral, according to the anatomic relationship between the cyst and the muscles of the floor of the mouth [1–10]. The floor of the mouth is the second most common site for dermoid cysts in the head and neck region after the lateral eyebrow.

The differential diagnosis of sublingual lesions includes infectious process, ranula, lymphatic malformation, heterotopic gastrointestinal cyst, and duplication foregut cyst [1–9]. For this reason, bimanual conventional radiography is not always sufficient for making a differential diagnosis. Computed tomography and magnetic resonance imaging allow more precise localization of the lesion in relationship to the geniohyoid and mylohyoid muscles, and they also enable the surgeon to choose the most appropriate surgical approach, especially in the case of very large lesions [1–9].

 Treatment is by enucleation via an intraoral or extraoral approach. An intraoral approach is recommended by most authors for sublingual cysts of small or moderate dimensions (less than 6 cm) above the mylohyoid muscle, whereas an extraoral approach is preferred for larger sublingual cysts (more than 6 cm) [11, 12]. In present case, the cyst was large (6.5 cm) and extending mylohyoid muscle, however, an intraoral approach was applied to avoid cosmetic problems. Appropriate traction and countertraction of the cyst might help to remove the cyst from mylohyoid muscle without extraoral incision. El-Hakim and Alyamani used an intraoral approach for large, deep-seated uninfected lesions, obtaining good aesthetics and function [11, 12]. The extraoral approach is used for very large dermoid cysts affecting the submandibular and submental spaces and in cases of infection that could compromise the patient's airway [11, 12].

 Recurrence is very rare with complete excision of the lesion, but a 5% rate of malignant transformation of oral dermoid cysts into the teratoid type has been reported in literature [13].

## 4. Conclusion 

Appropriate imaging techniques are necessary in the preoperative diagnosis of dermoid cysts of the mouth. Oral approach in surgical enucleation is useful procedure to avoid cosmetic problems in large and entending the mylohyoid muscle cyst.
